# Development and analytical validation of a 25-gene next generation sequencing panel that includes the *BRCA1* and *BRCA2* genes to assess hereditary cancer risk

**DOI:** 10.1186/s12885-015-1224-y

**Published:** 2015-04-02

**Authors:** Thaddeus Judkins, Benoît Leclair, Karla Bowles, Natalia Gutin, Jeff Trost, James McCulloch, Satish Bhatnagar, Adam Murray, Jonathan Craft, Bryan Wardell, Mark Bastian, Jeffrey Mitchell, Jian Chen, Thanh Tran, Deborah Williams, Jennifer Potter, Srikanth Jammulapati, Michael Perry, Brian Morris, Benjamin Roa, Kirsten Timms

**Affiliations:** 1 Myriad Genetic Laboratories, Inc., Salt Lake City, Utah USA; 2 Myriad Genetics, Inc., Salt Lake City, Utah USA

**Keywords:** Next generation sequencing, Hereditary cancer, *BRCA1*, *BRCA2*

## Abstract

**Background:**

Germline DNA mutations that increase the susceptibility of a patient to certain cancers have been identified in various genes, and patients can be screened for mutations in these genes to assess their level of risk for developing cancer. Traditional methods using Sanger sequencing focus on small groups of genes and therefore are unable to screen for numerous genes from several patients simultaneously. The goal of the present study was to validate a 25-gene panel to assess genetic risk for cancer in 8 different tissues using next generation sequencing (NGS) techniques.

**Methods:**

Twenty-five genes associated with hereditary cancer syndromes were selected for development of a panel to screen for risk of these cancers using NGS. In an initial technical assessment, NGS results for *BRCA1* and *BRCA2* were compared with Sanger sequencing in 1864 anonymized DNA samples from patients who had undergone previous clinical testing. Next, the entire gene panel was validated using parallel NGS and Sanger sequencing in 100 anonymized DNA samples. Large rearrangement analysis was validated using NGS, microarray comparative genomic hybridization (CGH), and multiplex ligation-dependent probe amplification analyses (MLPA).

**Results:**

NGS identified 15,877 sequence variants, while Sanger sequencing identified 15,878 in the *BRCA1* and *BRCA2* comparison study of the same regions. Based on these results, the NGS process was refined prior to the validation of the full gene panel. In the validation study, NGS and Sanger sequencing were 100% concordant for the 3,923 collective variants across all genes for an analytical sensitivity of the NGS assay of >99.92% (lower limit of 95% confidence interval). NGS, microarray CGH and MLPA correctly identified all expected positive and negative large rearrangement results for the 25-gene panel.

**Conclusion:**

This study provides a thorough validation of the 25-gene NGS panel and indicates that this analysis tool can be used to collect clinically significant information related to risk of developing hereditary cancers.

## Background

Approximately 7% of breast and 11% to 15% of ovarian cancers are estimated to be due to germline DNA mutations [1-3]. Mutations in the *BRCA1* and *BRCA2* genes account for the majority of the mutations that increase risk for these cancers [1-3]. In addition to these two genes, mutations in several others, such as *MLH1* and *MSH2,* can also convey significant increases in risk for the development of malignancies in other hereditary cancer syndromes [4]. The detection of germline mutations in blood samples from patients can be extremely useful for identifying patients at high risk of developing a malignancy. This genetic information can be used to guide treatment discussion and genetic counseling for at-risk family members.

Sanger DNA sequencing has been the standard method of screening for genetic variants in clinical practice. In order to use this methodology, users are required to focus on small groups of genes that are selected based on a patient’s unique risk factors and family history. Consequently, the utility of the Sanger method is limited when analyzing multiple genes from several patients simultaneously because tests for targeted genes often need to be conducted serially instead of simultaneously [5]. However, this upfront selectivity combined with incomplete follow-through in reflex testing may reduce the sensitivity of the testing overall.

Next generation sequencing (NGS) platforms provide an alternative to Sanger DNA sequencing that is more efficient for the analysis of large gene panels, allowing for more effective simultaneous screening of multiple genes [6]. This technique relies on multiplexed sample preparation followed by massive parallel sequencing and requires a significant informatics component for analysis [6]. Because multiple genes can be analyzed at once, delays in the acquisition of genetic data can be reduced [6]. Use of NGS could enable physicians to assess many genes associated with increased cancer risk at once, providing results in less time than required for several Sanger sequencing analyses to be conducted serially. However, a lack of standardization in sample preparation techniques, platforms, data analysis methods, variant classification, and clinical interpretation are significant challenges to the use of NGS platforms in clinical practice. To that end, guidelines and recommendations for NGS have been developed [7,8].

An optimized and validated assay design is critical to maximizing the analytical sensitivity and specificity of NGS assays and ensuring high-quality interpretation to facilitate clinical decision-making. The development and analytical validation of a clinical NGS panel of 25 genes associated with hereditary cancers that can be screened simultaneously for maximal efficiency is presented here.

## Methods

### Development of the 25-gene panel

Twenty-five genes associated with hereditary cancer syndromes [9] were selected for development of a panel to screen for syndromes associated with 8 primary types of cancer (breast, ovarian, colon, endometrial, melanoma, pancreas, gastric, and prostate) using NGS. The genes included in the panel were: *BRCA1, BRCA2* (hereditary breast and ovarian cancer syndrome); *MLH1, MSH2, MSH6, PMS2, EPCAM* – for large rearrangements of the last two exons only (Lynch syndrome); *APC* (familial adenomatous polyposis/attenuated familial adenomatous polyposis syndrome); *MUTYH* (*MUTYH*-associated colon cancer risk/*MUTYH*-associated polyposis syndrome); *CDKN2A* (melanoma-pancreatic cancer syndrome); *PALB2, ATM* (hereditary breast and pancreatic cancer risk); *STK11* (Peutz-Jeghers syndrome), *PTEN* (*PTEN* hamartoma tumor syndrome); *TP53* (Li-Fraumeni syndrome); *CDH1* (hereditary diffuse gastric cancer syndrome); *BMPR1A, SMAD4* (juvenile polyposis syndrome); *BARD1* (hereditary breast cancer risk); *CHEK2 (*hereditary breast, colorectal and prostate cancer risk); *CDK4* (melanoma cancer syndrome); *NBN* (hereditary breast and prostate cancer risk); *RAD51C, BRIP1* (hereditary breast and ovarian cancer risk); and *RAD51D* (hereditary ovarian cancer risk) (Table 1). Most of these genes are associated with medical management guidelines from professional societies, such as NCCN, but some have currently only been associated with higher lifetime risks in published studies and do not have associated medical management guidelines. Variants were classified using methods consistent with American College of Medical Genetics and Genomics Guidelines [10].

**Table 1:** **Genes included in the 25-gene NGS panel**

Gene name	Transcript ID
*APC*	NM_000038.5
*ATM*	NM_000051.3
*BARD1*	NM_000465.3
*BMPR1A*	NM_004329.2
*BRCA1*	NM_007294.3
*BRCA2*	NM_000059.3
*BRIP1*	NM_032043.2
*CDH1*	NM_004360.3
*CDK4*	NM_000075.3
*CHEK2*	NM_007194.3
*EPCAM*	NM_002354.2
*MLH1*	NM_000249.3
*MSH2*	NM_000251.2
*MSH6*	NM_000179.2
*MUTYH (alpha5)*	NM_001128425.1
*MUTYH (alpha3)*	NM_001048171.1
*NBN*	NM_002485.4
*P14ARF*	NM_058195.3
*P16*	NM_000077.4
*PALB2*	NM_024675.3
*PMS2*	NM_000535.5
*PTEN*	NM_000314.4
*RAD51C*	NM_058216.2
*RAD51D*	NM_002878.3
*SMAD4*	NM_005359.5
*STK11*	NM_000455.4
*TP53*	NM_000546.5

DNA target preparation and enrichment were performed using the RainDance microdroplet polymerase chain reaction (PCR) system (RainDance Technologies, Billerica, Massachusetts). The RainDance microfluidic system combines microdroplets containing DNA and PCR reaction mix with microdroplets containing PCR primer sets. The use of dropletized primer/DNA combinations allowed for use of high numbers of primers in the library without primer-primer interactions. Custom primers were then arranged into multiplexes of 5 amplicons for efficient DNA usage and dropletized into the RainDance library.

A custom primer library to amplify gene regions of interest was designed using an iterative process. Sequencing regions of coding exons were identified (Table 1) and flanked by up to 20 bases of upstream and 10 bases of downstream intronic sequence to allow for variants that occurred in conserved, proximal splicing elements. Several extensions to cover potential mutations such as more distal, putative splicing mutations or other non-exonic mutations were included in the design [11,12]. A list of all regions was assembled and provided to RainDance Technologies for automated primer design with required design criteria, in this case Illumina 2X150 base-pair paired end sequencing, to determine optimal amplicon length, primer placement and primer tail sequences. Putative PCR priming sites were selected by RainDance Technologies using genome build hg19/GRCh37 with custom software and compared automatically with public variant databases to avoid nonspecific priming and common single nucleotide polymorphisms. Primers which were designed against genes that are currently tested in this laboratory by Sanger sequencing were also manually compared against our own variant lists to avoid common sequence variations that might interfere with primer binding. The risk of sequence artifacts due to interfering variants at primer binding sites is reduced through amplicon tiling in all but the terminal primers of gene regions. With an average of >5 amplicons per exon there is enough redundancy for most sequence variants that might alter primer bindings in other amplicons to be detected. This library was then synthesized and tested for quality and reproducibility. Shortcomings were addressed through new primer design before the process was repeated.

**Figure 1:**
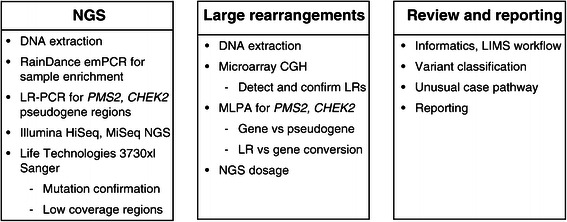
**Components of the 25-gene panel.** CGH, comparative genomic hybridization; emPCR, emulsion polymerase chain reaction; LIMS, laboratory information management system; LR, large rearrangement; MLPA, multiplex ligation-dependent probe amplification, NGS, next generation sequencing.

This assay can be broadly divided into 3 parts: the sequencing portion via NGS; the large rearrangement (LR) detection via NGS dosage analysis, microarray CGH, and multiplex ligation-dependent probe amplification analyses (MLPA); and informatics assisted data review and reporting, including variant classification (Figure 1). For the NGS assay, genomic DNA was extracted from blood samples by QIAsymphony using the DSP DNA Midi kit (Qiagen, Venlo, The Netherlands), and a fixed input of 5 μg was fragmented to approximately 3 kb using sonication (SonicMan, Brooks Life Science Systems, Spokane, Washington). The reaction mix containing fragmented DNA and PCR mastermix was dropletized and merged with the droplets containing the primers through a high throughput microfluidic emulsion PCR system using a RainDance Thunderstorm. The fragmented, genomic template for this step is limited so that amplification is digital for most reactions; roughly 40,000 reaction droplets were inoculated per patient to ensure that the final PCR products were consistent and normalized. The resulting emulsion underwent 55 cycles of amplification on a Mastercycler Pro thermocycler (Eppendorf, Hamburg, Germany) after which the PCR products were bead-purified using AMPure XP (Beckman Coulter, Brea, California). A 6-nucleotide “barcode” tag, specific to each sample within a batch, and Illumina-specific sequencing adaptors were attached using secondary PCR. Purified products were then pooled and sequenced on the Illumina HiSeq 2500 NGS instrument (Illumina Inc., San Diego, California) to generate 2X150 base-pair paired end sequencing reads according to manufacturer instructions. All reagents were stored according to and used within the timeframe specified by manufacturer recommendations.

Portions of the *PMS2* and *CHEK2* genes have highly homologous pseudogenes. Therefore, target enrichment was modified to incorporate long-range PCR to ensure specificity to the genes of interest. Specific long-range amplicons (LRAs) were generated by primary PCR performed using LA Taq Hot Start (Takara Bio Inc., Otsu, Japan) on 50 ng of genomic DNA. LRA products were diluted 1:10,000, and a second round of PCR was completed to amplify specific regions of interest and to attach barcode and sequencing adaptors to samples. Equal amounts of sample were combined and diluted to 2 nM for sequencing using the Illumina MiSeq NGS instrument for 2X150 base-pair paired end sequencing reads. These data for individual patients generated on the HiSeq and MiSeq are recombined informatically during data analysis.

### NGS informatic data analysis

Base calling was completed using Illumina Sequence Control Software with Real Time Analysis. Samples had quality scores assigned, and all sequence reads were trimmed to remove primers and sequencing bases below Q30 using an optimized Burrows-Wheeler approach. The open-source program JAligner (http://jaligner.sourceforge.net) was used to align the trimmed sequence reads to an internal set of reference sequences comprising the genes of this panel and their associated pseudogenes. The system automatically discarded all reads that matched pseudogene reference sequences as well as or better than the genes of interest.

**Figure 2:**
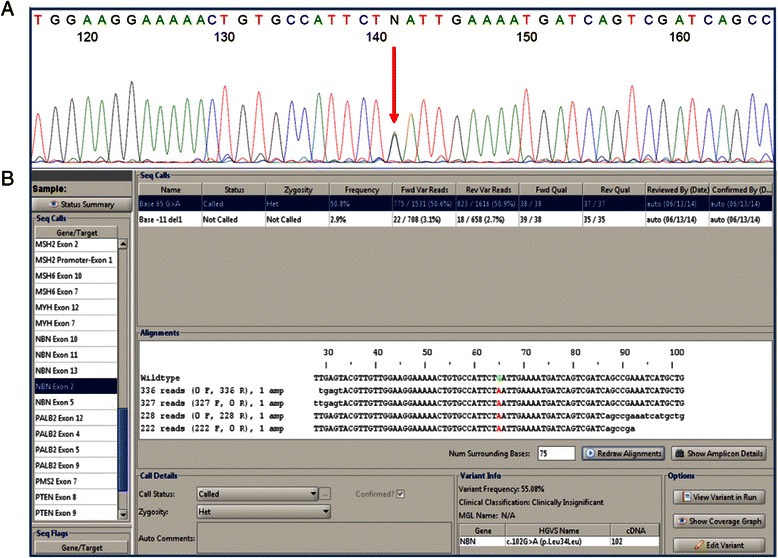
**The same variant in*NBN*as detected by Sanger sequencing (A) and NGS (B).** Note that the frequency of alleles at variable positions, read depth in independent forward and reverse reads and quality scores can be reviewed here. The heterozygous base change indicated by the arrow in panel **A** is the same base change selected in the NGS results in panel **B**. *NBN*, *NBN*-associated cancer risk ; NGS, next generation sequencing.

All data were reviewed using in-house–designed review software. This software annotated allele frequency, assessed zygosity and performed quality metrics. To assess quality, Q scores and percent mapped reads were calculated. Using this method, the average depth of coverage was >1000X, with a minimum depth of coverage of 50X per base. By maintaining the average depth of coverage around 1000X to 2000X the number of bases with low (50X to 100X) coverage can be minimized. High depth of coverage becomes important when trying to assess dosage by amplicon quantitation as a method for detecting large rearrangements. Any region covered by NGS sequencing with a depth of coverage <50X was repeated using Sanger sequencing. After alignment, sequence variants were called based on quantitative thresholds; bases called with a non–wild-type frequency of <10% are attributed to noise, 30% to 70% are called heterozygous changes, and 90% to 100% are called homozygous changes (Figure 2). Bases in intermediate frequencies (10%-30%, or 70%-90%) are followed up by Sanger sequencing.

### Analysis of large rearrangements

**Figure 3:**
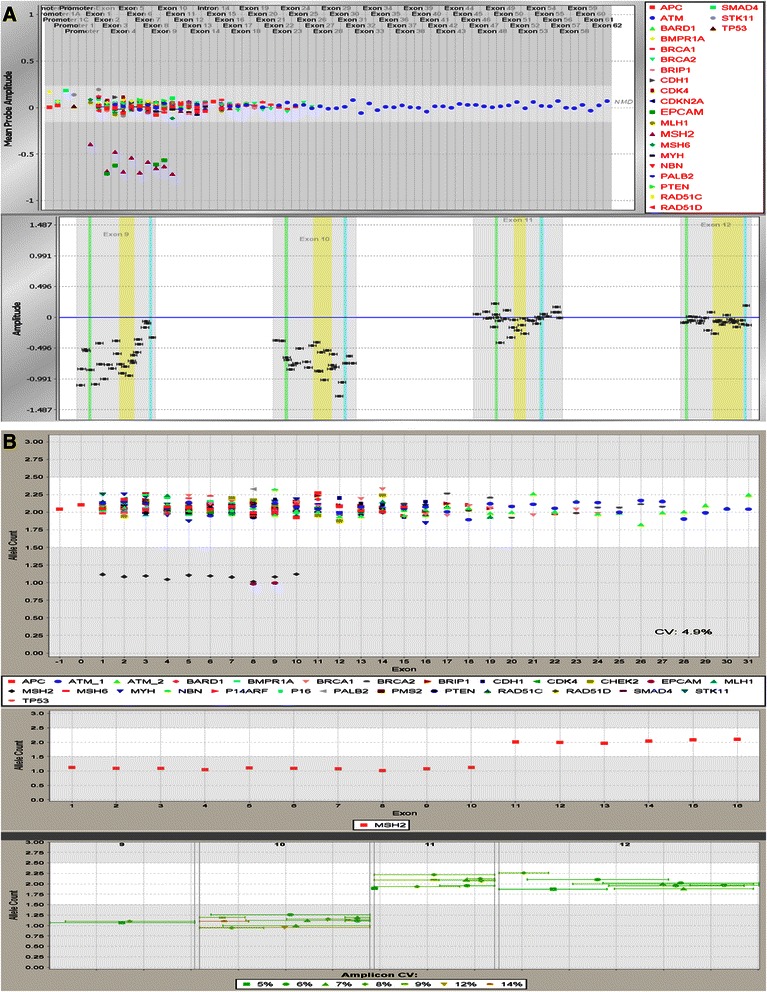
**A contiguous deletion involving*EPCAM*and*MSH2*as seen in both microarray CGH (A) and NGS LR (B) views.** In the case of the microarray CGH plot the data are on a log_2_ scale with wild-type dosage at 0 on the Y-axis. In the case of the NGS dosage plot, the data are on a linear scale with wild-type dosage at 2 on the Y-axis. In both cases, a summary overview is available (top) as well as a zoomed in (bottom) view showing specific probe or amplicon placement. In the summary view, all genes are shown simultaneously with a symbol representing each gene, and exons proceeding 5’ to 3’ across the X-axis. Note that *EPCAM* exons 2–3 are also included in the microarray CGH assay for normalization purposes but are not tested on the NGS LR assay. NGS LR, next generation sequencing large rearrangements.

Large rearrangements were identified using quantitative dosage analysis of the data obtained from NGS. This approach relied on the digital nature of the droplet PCR process and required the comparison of trimmed, mapped amplicon read counts for all 96 samples in a batch. For these data, read counts of each amplicon were first normalized to the average read count of the sample. This ratio was then normalized to adjust for variability across the batch of 96 samples. Next, all of the amplicons that overlapped an exon or region of interest were combined together into a summary value. Finally, a ratio for each region of interest was generated for each sample relative to all others within the batch and plotted as a scatterplot. This analysis was done using in-house–developed review software by trained data reviewers (Figure 3).

Identification of large rearrangements, specifically deletions and duplications, was also performed using additional methods to complement NGS. Twenty-three genes in the panel were assessed using a custom microarray comparative genomic hybridization (CGH) chip (Agilent Technologies, Santa Clara, California). A total of 349 regions, mainly coding exons along with 100 nucleotides of flanking sequences, were covered by >9,400 custom oligonucleotide probes for an average coverage of 27 probes per region. Patient genomic DNA was fragmented and labeled with Cy5, and reference DNA was labeled with Cy3 using a custom version of the Agilent SureTag Labeling Kit. Labeled DNA was then combined and hybridized to a microarray containing oligonucleotide probes to gene regions. After hybridization, slides were washed to remove excess hybridization reagents and non-hybridized DNA. Scanning of washed slides was completed using an Agilent Microarray Slide Scanner, and data were extracted using Agilent Feature Extraction Software. The analysis was performed by in-house–developed software using sample dosage normalization, locally weighted scatterplot smoothing normalization, historic probe normalization, and custom GC normalization (Figure 3).

MLPA analysis for large rearrangements in *PMS2* and *CHEK2* was conducted using probe mixes P008 and P190, respectively, from MRC-Holland (Amsterdam, The Netherlands). Pairs of adjacent oligonucleotide probes were hybridized to regions of interest, usually one probe pair in or near each exon. To improve specificity, probes were designed over gene-specific variants, to distinguish between homologous pseudogenes and the actual gene regions of *PMS2* and *CHEK2.* Adjacent probes were ligated and then amplified by PCR using fluorescently labeled composite primers that included genomic target and stuffer sequences to differentiate products by size. Gene dosage results were analyzed using GeneMarker software (Softgenetics, State College, Pennsylvania).

### Validation procedures

The 25-gene panel was validated using samples submitted for single-syndrome clinical testing to a Clinical Laboratory Improvement Amendments (CLIA) and College of American Pathology (CAP) approved laboratory. All patients who received clinical testing gave informed consent for testing and were over the age of 18. Upon completion of clinical testing, all samples were anonymized for research by Myriad’s Quality Assurance department. Any samples originating from states with legislation mandating destruction of biospecimens after completion of genetic testing were excluded. As a retrospective study performed on anonymized samples, this analytical validation was not subject to any additional review (HHS regulation 45 CFR 46). The sequencing component of the NGS panel was validated by comparing NGS with Sanger sequencing results in 100 samples. To facilitate this, Sanger primer sets were designed and tested for all exons sequenced by the NGS panel.

Large rearrangement analysis components of the 25-gene panel were validated on additional positive samples, along with the 100 samples used for the sequencing portion of the validation which did not contain large rearrangement mutations. Deletion/duplication analyses on 23 genes were also validated by microarray CGH in 212 anonymized DNA samples and by NGS dosage analysis on a subset of 110 of these anonymized samples with sufficient volume. Genomic positive controls were supplemented with synthetic controls for microarray CGH. MLPA was validated for *PMS2* and *CHEK2* in 110 anonymized DNA samples. This set of 110 anonymized samples is the same set that was used in the microarray CGH and NGS dosage analysis validations.

### Statistical methods

Sensitivity is the proportion of the number of true positives over the sum of the number of true positives and false negatives. It is the ability to correctly identify a sequence change from the wild type if that change exists. Specificity is the proportion of the number of true negatives over the sum of the number of true negatives and false positives. It is the ability to correctly exclude a sequence change from the wild type if that change does not exist.

Lower 95% confidence bounds for sensitivity and specificity estimates were calculated using Minitab version 15, 1 proportion test, with the Exact method. Note that Minitab only calculates a 1-sided confidence interval (CI) when the numerator of the proportion equals the denominator because the upper bound is 100%. By reporting the lower limit of the 95% CI, one can claim with 95% confidence that the actual sensitivity or specificity meets or exceeds the stated sensitivity or specificity.

## Results

### Initial sensitivity and specificity assessment of *BRCA1* and *BRCA2* sequencing

For the initial sequencing assessment, NGS identified 15,877 variants, while prior Sanger sequencing identified 15,878 variants among 1864 anonymized samples from patients who had previously undergone *BRCA1* and *BRCA2* testing. Patients were selected based on personal history that included cancer, though not limited to breast or ovarian malignancies*.* Of these variants, 3.02% were deleterious or suspected deleterious mutations while only 0.67% were variants of uncertain significance. The results showed an estimated analytical sensitivity for NGS >99.96% (lower limit of the 95% CI) and an estimated analytical specificity >99.99% (lower limit 95% CI) for *BRCA1* and *BRCA2* sequencing. One polymorphism, *BRCA2* c.7806-14C > T, was missed by NGS, determined to be caused by non-amplification of a PCR allele. The missed polymorphism was in a DNA sample with a rare co-occurring 3-base intronic deletion in a PCR primer binding site (*BRCA2* c.7806-64_7806-62del), which has a frequency of approximately 0.05%. No other differences in mutations detected were observed, including the 301 indels and 15,577 single-base substitutions identified here, or sensitivity for different types of mutations between Sanger sequencing and NGS.

Sensitivity of subsequent NGS data analysis was enhanced by using individual amplicon dosage to assess PCR allele dropout due to rare sequence variants under primer binding sites. First, the dosage of each amplicon relative to the rest of the amplicons in the sample is examined. Next the dosage is compared with the standard deviation of the amplicon across the whole batch. Based on these data, any amplicon that appears amplified from a single allele can be flagged. Flagged regions can be assessed through follow-up Sanger sequencing. This improvement was made possible by the enhanced quantitative nature of NGS that was not an option with Sanger sequencing. PCR-based sample preparation for both NGS and Sanger sequencing shares the same risk of variants interfering with primer binding but NGS allows for better detectability. Based on these initial positive results, the 25-gene NGS panel underwent further validation.

### Analytical validation of the 25-gene NGS panel for clinical testing

At this point changes to the assay were completed and validation was performed for the sequencing and large rearrangement components for the entire 25-gene panel test. NGS and Sanger sequencing were performed in parallel on 100 anonymized DNA samples. Sequencing results were 100% concordant for the 3923 collective variants identified in 100 DNA samples (Table 2). This included 3884 single base substitutions and 39 indels. The 39 indels included 4 insertions, 34 deletions, and one insertion coupled with a deletion. Of these, 20 occurred in coding exons and 19 occurred in intronic regions flanking the exons that are involved in splicing. Analytical sensitivity of the NGS assay was estimated to be >99.92% (lower limit of 95% CI). Analytical specificity of the NGS assay was estimated to be >99.99% (lower limit of 95% CI). Reproducibility studies were also performed, wherein 4 DNA samples were run in triplicate per batch, across 3 different batches. The data showed 100% concordant calls, which demonstrated intra-batch and inter-batch reproducibility.

**Table 2:** **Sanger and next generation sequencing results**

	Sanger sequencing	NGS
Samples	100	100
Amplicons (per sample)	370	1969
Bases analyzed (per sample)	88,631	88,631
Total positive bases	3923	3923
•Single base substitutions	3884	3884
•Small indels	39	39
Total negative bases	8,859,177	8,859,177

The large rearrangement component of the assay was validated using an additional set of 212 anonymized DNA samples with known large rearrangement genotypes. Deletion/duplication analysis on 23 of the 25 genes in the panel was performed by microarray CGH, which correctly identified all 51 genomic positive controls, including replicates and reproducibility controls, across different genes among the 212 anonymized DNA samples (Table 3). A partial set of 110 of these samples with sufficient volume, including 49 of the positive samples, was also processed using NGS for large rearrangement dosage analysis. Of the 49 large rearrangement positive samples processed, 48 produced results which were all concordant with the expected sample large rearrangements. The sample containing the final LR, a *MSH2* deletion of exons 1–6, did not successfully complete laboratory processing and did not undergo data analysis. For some genes where rare genomic positive controls were not available, we supplemented the validation studies with synthetic positive controls created with restriction enzyme digests of genomic DNA for microarray CGH analysis. The results were consistent with simulated deletions in the affected regions. In addition, deletion/duplication analysis for the pseudogene-containing *PMS2* and *CHEK2* genes was validated using MLPA on 110 anonymized DNA samples with known genotypes. MLPA correctly identified all 5 genomic positive controls in *PMS2* and *CHEK2* among these 110 anonymized DNA samples.

**Table 3:** **Previously characterized large rearrangements that were included in the study**

Gene	Mutation	microarray CGH	NGS dosage analysis	MLPA
*APC*	deletion promoter 1B - exon 3	detected	detected	*n/a*
	deletion exon 4	detected	detected	*n/a*
*BRCA1*	duplication promoter 1A - exon 2	detected	detected	*n/a*
	deletion promoter 1A - exon 19	detected	detected	*n/a*
	duplication exons 5 - 7	detected	detected	*n/a*
	deletion exon 13	detected	*n/a -- insufficient volume*	*n/a*
	duplication exon 13	detected	detected	*n/a*
	deletion exons 15 – 16	detected	*n/a -- insufficient volume*	*n/a*
	deletion exons 16 - 17	detected	detected	*n/a*
	deletion exon 24	detected	detected	*n/a*
*BRCA2*	duplication exons 12 - 13	detected	detected	*n/a*
*EPCAM*	deletion exons 2 - 3' UTR	detected	detected	*n/a*
*MLH1*	deletion exons 4 - 9	detected	detected	*n/a*
	deletion exon 13	detected	detected	*n/a*
	deletion exon 14	detected	detected	*n/a*
	deletion exon 16	detected	detected	*n/a*
*MSH2*	deletion exon 1	detected	detected	*n/a*
	deletion exons 1 - 6	detected	*No Result*	*n/a*
	duplication exons 1 - 6	detected	detected	*n/a*
	deletion exons 1 - 7	detected	detected	*n/a*
	deletion exons 1 - 8	detected	detected	*n/a*
	deletion exons 1 - 10	detected	detected	*n/a*
	deletion exons 8 - 15	detected	detected	*n/a*
	deletion exon 16	detected	detected	*n/a*
*MSH6*	deletion exons 4 (3' end) - 10	detected	detected	*n/a*
*MUTYH*	duplication exon 1	detected	detected	*n/a*
*PMS2*	deletion exon 10	*n/a*	*n/a*	detected
	deletion exons 1 - 5	*n/a*	*n/a*	detected
	deletion exons 13 - 14	*n/a*	*n/a*	detected
	deletion exons 14 - 15	*n/a*	*n/a*	detected
	deletion exons 9 - 11	*n/a*	*n/a*	detected

## Discussion

Recent advances in NGS and sample enrichment technologies allow for simultaneous assessment of multiple genes. Hereditary cancer panels have been constructed incorporating genes underlying well characterized cancer syndromes, such as *BRCA1* and *BRCA2*, along with more recently discovered genes associated with increased cancer risk [13]. The use of gene panels in hereditary cancer risk assessment is increasing and studies to assess the prevalence of mutations among patients commonly referred for genetic testing have recently been published [9,14,15]. Additional studies are underway to more fully define the benefits and limitations of panel testing in the clinical setting. Analytical validation is a critical step in the laboratory development process to facilitate the availability of highly robust and reproducible clinical tests. The current study demonstrates the analytical validation of a 25-gene hereditary cancer panel by comparing results of Sanger sequencing and other methods to results of the NGS assay.

Initial analysis of *BRCA1* and *BRCA2* gene variants facilitated the optimization of this NGS assay that allowed for validation of a comprehensive 25-gene panel. Process improvements in NGS data analysis based on these initial results were able to enhance the sensitivity of the assay for detection of low coverage regions that were suggestive of potential deletions or PCR allele dropouts. This initial analysis also supported the standardized NGS methodology so that it could then be applied to the collection of mutation data that could be clinically significant using the 25-gene panel. This is important because NGS is being used clinically, but there continue to be challenges in standardizing the different components that include the analytical wet bench process (sample preparation, target enrichment, NGS sequencing) and the bioinformatics pipeline for NGS [7]. Therefore, it is important for each lab to demonstrate a robust validation of their clinical NGS assays.

The validation of the gene panel presented here offers the opportunity to test a system that incorporates laboratory information management system tracking for all steps through the lab and custom software to support timely and accurate analysis of clinical data. Furthermore, it allowed testing and validation of a system for handling large numbers of novel variants requiring review and classification, and a comprehensive reporting system to meet the needs of patients and their health care providers.

The validation study showed that the 25-gene panel meets the rigorous quality standards necessary to provide useful data in the clinical setting. The results of the 25-gene panel were shown to be equivalent to those obtained using Sanger DNA sequencing analysis. Extensive validation covered the sequencing and large rearrangement components of the assay with suitably large study sets to provide reliable data.

The comprehensiveness of this assay was confirmed by leveraging multiple orthogonal methods during the validation. For example, sequencing variations were confirmed with Sanger sequencing (Figure 2) and NGS dosage analysis results were confirmed by microarray CGH (Figure 3). Though improvements in this process may still be made, the NGS system for detection of large rearrangement yielded 100% concordant results for all the large rearrangements in regions covered by both the RainDance and microarray CGH assays. Furthermore, the ability to assess dosage of amplicons allows users to catch some cases of allelic dropout that could be caused by sequence variants under terminal primers which would not be observable in overlapping amplicons. There are numerous potential advantages to LR detection via NGS. In addition to lowering the amount of DNA required for multiple assays and, therefore, requiring fewer redraws for patients, it also allows for very specific amplicon placement. This is important when dealing with pseudogenes, which are present in many of the genes in this panel, and means that dosage data from the actual coding bases of interest is retrieved rather than relying on nearby, and often intronic, divergent sequences. Finally, the PCR-based nature of the enrichment could also allow for the detection of Alu insertions that disrupt the PCR amplicons used for enrichment, and cause them to either fail to amplify or to amplify poorly. This is important as Alu insertions have been observed in coding regions of many genes and even occur as founder mutations such as the Alu insertion *BRCA2* exon 3 (c.156_157insAlu) in Portuguese populations [16]. These mutations are generally not detected by methods such as microarray CGH and MLPA. In addition, large rearrangements in *PMS2* and *CHEK2* were also assessed using MLPA, and non-pseudogene portions were confirmed against NGS and microarray CGH. This concordance between orthogonal methods confirms the strength of this testing platform.

There are some limitations to the study presented here. First, the validation was limited to blood-derived samples. This restricted, at least initially, the availability of the test to one sample type. In addition, rare variants such as deep intronic sequence changes, and rare large rearrangements such as genomic inversions, may not be identified using the PCR-based target enrichment approach used in this assay.

Use of this gene panel can help define a patient’s cancer risk and define management options, including changes in routine surveillance procedures at a cost that is comparable to that of single gene testing [17,18]. Importantly, the genes in the panel were selected because they could provide clinically significant data, not just an assessment of risk. The availability of a robust assay for these genetic risk factors that uses a standardized analysis procedure such as the one presented here can facilitate more widespread screening for hereditary cancer syndromes.

## Conclusions

These findings represent a thorough validation of the 25-gene hereditary pan-cancer panel. The results demonstrate that the NGS panel can be used to screen patients for mutations associated with hereditary cancers in a variety of different tissues with a sensitivity and specificity comparable to that of Sanger sequencing. The NGS panel has the advantage of being able to simultaneously screen for multiple genes from several different patients at the same time. Widespread use of this standardized genetic risk assessment tool could increase the identification of patients at high risk for these cancers and potentially improve care by changing surveillance procedures and/or treatment of malignancies.

## Abbreviations

CGH: Comparative genomic hybridization
CI: Confidence interval
LR: Large rearrangements
LRA: Long-range amplicons
MLPA: Multiplex ligation-dependent probe amplification analyses
NGS: Next generation sequencing
PCR: Polymerase chain reaction
